# Duration of Diabetes as a Risk Factor for Retinal Microvasculature Alterations Detected with Optical Coherence Tomography Angiography in Patients without Clinical Retinopathy

**DOI:** 10.3390/diagnostics12123020

**Published:** 2022-12-02

**Authors:** Jing Qian, Zeeshan Haq, Daphne Yang, Joy Q. Jin, Jay M. Stewart

**Affiliations:** 1 Department of Ophthalmology, University of California, San Francisco, CA 94158, USA; 2 Department of Ophthalmology, Zuckerberg San Francisco General Hospital and Trauma Center, San Francisco, CA 94110, USA; 3 Children’s Hospital of Nanjing Medical University, Nanjing 210008, China

**Keywords:** optical coherence tomography angiography, diabetes, foveal avascular zone, superficial capillary plexus, vessel density

## Abstract

In this study, we examined the impact of diabetes mellitus (DM) disease duration on optical coherence tomography angiography (OCTA) parameters in diabetic patients without diabetic retinopathy (DR). A total of 1118 eyes from 1118 DM patients without DR were divided into three groups by DM duration: 0–5 years (short cohort; *n* = 571), 6–10 years (medium cohort; *n* = 306), and >10 years (long cohort; *n* = 241). Ultra-widefield fundus photography and nine OCTA parameters derived from the superficial retinal capillary plexus were analyzed. Perfusion density (PD) and vessel length density (VD) were significantly decreased within the 1 mm patch in patient OCTAs from the medium cohort compared to the short cohort. Conversely, PD and VD were significantly decreased within the 6 mm patch and inner ring among the long cohort compared to the short and medium cohorts. These findings remained consistent after controlled analysis. Patients in the medium cohort had the largest FAZ area, while patients in the long cohort had the smallest FAZ area, with a statistically significant difference between the two groups. Superficial PD and VD significantly decreased among the medium and long cohorts compared to the short cohort, confirming that subclinical, progressive macular vasculature change is associated with longer DM duration. However, while FAZ area significantly increased in the medium cohort, the long cohort exhibited decreased FAZ area, suggesting the latter may possess protective factors that decrease overall risk of DR development.

## 1. Introduction

Diabetic retinopathy (DR) is a common microvascular complication of diabetes mellitus (DM) and is the leading cause of preventable blindness in working aged people [1]. In the past, fluorescein angiography (FA) has been the gold standard for the evaluation of DR [2]. However, FA is time-consuming and requires invasive intravenous dye administration. Furthermore, it is not routinely performed, especially for eyes without visible retinopathy or at an early stage of DR [3]. Optical coherence tomography angiography (OCTA) has emerged as a relatively new, non-invasive imaging technique that can provide rapid and high-resolution images of the retinal microvasculature as well as quantitative data without the injection of dye [4,5].

Microaneurysm formation is the most common early clinically visible manifestation of DR [6]. It was reported that OCTA could detect microaneurysms in diabetic eyes without clinical DR [7]. Recent studies have also demonstrated that diabetic patients without clinically apparent DR have enlarged foveal avascular zones (FAZ) [8,9,10,11] and reduced vessel density [11,12] on OCTA compared to healthy subjects. Thus, OCTA is able to identify features of preclinical retinopathy that are not detectable by clinical examination.

Previous epidemiological studies have identified the duration of diabetes as a strong predictor for the progression of DR [13,14]. Interestingly, recent studies have also suggested that while duration of DM is generally associated with progression of end-stage complications such as DR, certain subgroups of patients may possess protective genetic or environmental factors that can contribute to the slowing or prevention of such complications [15]. It is unknown whether quantitative OCTA parameters change with prolonged diabetes duration. The aim of this study was to assess the impact of disease duration on OCTA parameters in diabetic patients with no clinical DR.

## 2. Materials and Methods

### 2.1. Subjects

This cross-sectional study retrospectively reviewed imaging obtained from diabetic subjects who presented for retinopathy screening examinations at Zuckerberg San Francisco General Hospital and Trauma Center between April 2018 and March 2020. Ultra-widefield fundus photography (Optos Daytona; Optos PLC, Dunfermline, UK) and OCTA were obtained for all patients. Fundus photographs were evaluated by the department’s DR screening program reading center and graded for retinopathy severity. All eyes classified as having DM without DR were considered for inclusion in the study. Exclusion criteria included any history of significant ocular injury or any ocular disease that could affect the retinal microvasculature such as retinal vascular occlusion, glaucoma, or vitreomacular disease, as such conditions have also been noted to show subclinical OCTA changes [16,17].

Relevant demographic and clinical information including age, sex, duration of DM, use of DM medications, most recent hemoglobin A1c, body mass index (BMI), and presence of comorbid disease (e.g., hypertension, hyperlipidemia) were recorded. In accordance with prior work, DM disease duration was determined by patient self-report and/or medical record review [18,19,20]. An overwhelming majority of patients had type 2 diabetes. Patients were divided into three groups depending on the duration of their diabetes: 0–5 years (short cohort), 6–10 years (medium cohort), and more than 10 years (long cohort).

Institutional review board approval was granted by the University of California, San Francisco Human Research Protection Program (HRPP). All research activities adhered to the tenets of the Declaration of Helsinki. The UCSF HRPP granted a waiver of informed consent due to the retrospective nature of this study.

### 2.2. OCTA Image Acquisition and Analysis

All participants underwent OCTA imaging on a Cirrus HD-OCT 5000 with AngioPlex OCT Angiography (Carl Zeiss Meditec, Dublin, CA, USA) with 6 mm × 6 mm and 3 mm × 3 mm fields of view centered at the fovea. OCTA scans were comprised of 245 clusters of B-scans repeated four times, in which each B-scan consisted of 245 A-scans. The effect of eye motion-related artifacts was minimized using FastTrac eye tracking software [21]. Images were evaluated at the time of acquisition for quality. Satisfactory images to be included in the study were required to have a signal strength greater than 7, minimal motion artifacts, decentralization from the foveal center of less than 20 µm, and minimal evidence of obscuration by media opacities, as described previously [22]. In this study, one eye was selected for inclusion from each patient. If both eyes from an individual patient had images of equal quality, the right eye was included in the study.

Image analysis was automatically performed by the Cirrus AngioPlex embedded software, which provided automated segmentation and calculation of retinal microvascular metrics. The analysis was limited to the inner retina, which was defined as the tissue between the inner limiting membrane and an anterior offset from the retinal pigment epithelium of 110 μm. The superficial capillary plexus (SCP) was defined as the superficial 70% of the inner retina, and the deep capillary plexus (DCP) was the remaining 30% of the inner retina [23]. At present, the software only automatically reports values for the SCP; therefore, only SCP parameters were included.

**Figure 1:**
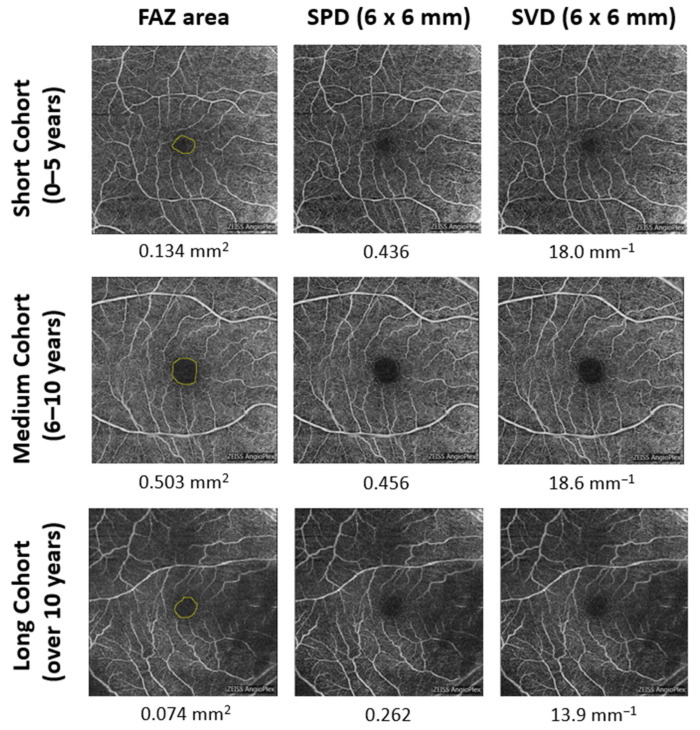
OCTA image acquisition from superficial capillary plexus. Each row shows a representative participant from each group: short cohort (0–5 years DM duration), medium cohort (6–10 years DM duration), and long cohort (greater than 10 years DM duration). Each column shows the quantitative OCTA parameters: FAZ area, SPD in the 6 mm patch, and SVD in the 6 mm patch. The yellow circle in the first column outlines the FAZ border.

OCTA parameters derived from the SCP were classified into two categories: (1) FAZ-related metrics, consisting of FAZ area, FAZ perimeter, and FAZ circularity index (CI); and (2) vessel density-related metrics, consisting of superficial perfusion density (SPD) and superficial vessel density (SVD) in the 6 mm patch (6 mm diameter around the foveal center), 1 mm patch (1 mm diameter around the foveal center), and inner ring (1 mm patch excluding the FAZ) [10,22]. Cirrus 11.0 software automatically calculated FAZ-related, SPD, and SVD metrics (Figure 1).

### 2.3. Statistical Analysis

Statistical analysis was performed using SPSS software 23 (SPSS, Inc., Chicago, IL, USA) and GraphPad Prism 8 (Windows, GraphPad Software, La Jolla, CA, USA). The Shapiro–Wilk test was used to determine whether the data were normally distributed. Comparisons between three or more groups were made using the Kruskal–Wallis H test or one-way ANOVA test. Differences between two groups were estimated with a Mann–Whitney *U* test or *t*-test. Multivariate linear regression models were used to analyze potential associations between each OCTA parameter and systemic risk factors. All *p* values were based on two-tailed testing and considered to be statistically significant if *p* < 0.05. A Bonferroni correction was applied when appropriate.

## 3. Results

A total of 1118 patients with DM without DR were included in the study. The duration of DM ranged from a minimum of 0 years (less than 1 year) to a maximum of 51 years. The mean duration of diabetes was 6.98 ± 6.39 years.

A summary of the characteristics for each diabetes duration group is shown in Table 1. The difference in age was statistically significant between all three groups, and patients with a longer duration of diabetes were, on average, older. Patients with more than six years of diabetes had significantly higher HbA1c, higher prevalence of hypertension and hyperlipidemia, more diet control, and less insulin use compared to those with fewer than five years of disease.

**Figure 2:**
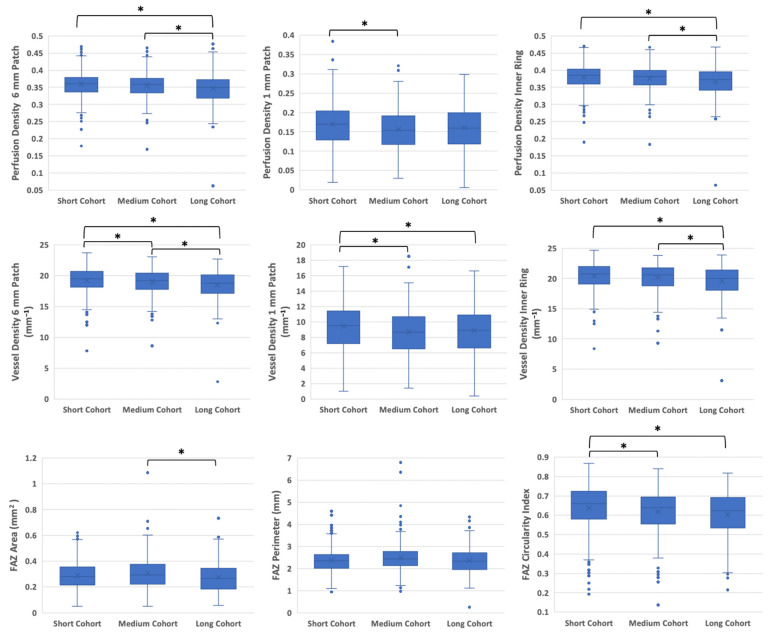
Graphs of density and foveal avascular zone metrics in the superficial capillary plexus (SCP) for the three diabetes duration groups. Boxes represent the interquartile range in each group. * *p* < 0.05.

Summary statistics for each OCTA parameter stratified by study group are shown in Table 2 and Figure 2. The SPD 6 mm patch, SPD inner ring, SVD 6 mm patch, and SVD inner ring parameters were significantly different between patients with a diabetes duration of over 10 years (long cohort) and those with fewer than 10 years. Patients in the 6–10-year cohort (medium cohort) had the lowest SPD 1 mm patch and SVD 1 mm patch, while the long cohort had increased averages for SPD and SVD 1 mm patch compared to the medium cohort. Significant differences for SPD 1 mm patch and SVD 1 mm were observed between patients with a diabetes duration of 0–5 years (short cohort) and those in the medium cohort. Patients in the medium cohort had the largest FAZ area, while patients in the long cohort had the smallest FAZ area, with statistically significant differences between the two groups. For FAZ circularity index, significant differences were observed between patients in the short cohort and those with more than five years of DM.

Table 3 shows the results of multivariate linear regression analysis. There were strong associations between age and all OCTA parameters except FAZ perimeter. In addition, there were strong associations between sex and SPD 1 mm patch, SVD 1 mm patch, FAZ area and FAZ perimeter. A diabetes duration of 6–10 years was significantly associated with SPD 1 mm patch, SVD 1 mm patch, and FAZ perimeter. A diabetes duration of over 10 years was weakly associated with SPD inner ring.

## 4. Discussion

Blindness is largely preventable in patients with DM with early diagnosis and intervention. FA is not routinely suggested for eyes without visible retinopathy or mild DR [3], but OCTA, as an alternative noninvasive modality, can be used to image retinal microvascular changes in diabetic patients before clinically visible retinopathy develops [8]. The thresholds for abnormal parameter values in previous OCTA studies were determined empirically by comparison with healthy eyes [8,9,10,11,12,24]. In the present study, we compared OCTA parameters in patients with DM with varying disease duration without retinopathy in order to identify the presence of any subclinical microvascular changes accompanying prolonged disease duration.

Our results support two intriguing concepts. First, we highlight quantitative OCTA parameters with significant differences between the 0–5-year DM cohort (short cohort) and the 5–10-year DM cohort (medium cohort), which suggest that these parameters may serve as novel markers for identifying subclinical progression towards DR after initial DM diagnosis. Second, the patient cohort with a DM duration of over 10 years (long cohort) did not exhibit significant changes in several OCTA parameters, suggesting that individuals who have been DR-free for an extended period since DM diagnosis may represent a relatively protected phenotype that is at lower risk of developing certain types of microvascular changes relevant to DR.

We propose several quantitative OCTA parameters not previously reported to indicate subclinical DR progression. When comparing the medium cohort against the short cohort, perfusion density (PD) and vessel density (VD) OCTA parameters in the 1 mm patch are significantly reduced. This suggests that the earliest signs of retinal change in patients on a trajectory to develop DR may begin at the fovea. In contrast, we observe a different pattern when comparing the long cohort against both short and medium cohorts. PD and VD in the 6 mm patch and inner ring are significantly reduced among the long cohort, while there is marginal increase with no significant difference in the 1 mm patch. This suggests that with extended diabetes duration in patients who have still not developed clinical retinopathy, microvascular changes become manifest in more peripheral locations. The PD and VD OCTA findings were significant even after controlled analysis, which suggests that PD and VD may be the earliest detectable DR microvascular changes related to disease duration. Interestingly, a prior study conducted by Cao et al. found that the vessel densities of SCP and DCP were not associated significantly with diabetes duration [12]. Furthermore, a prospective cross-sectional study performed by Durbin et al. consisting of 25 control subjects and 26 diabetic patients did not detect a correlation between vascular density or FAZ metrics and diabetes duration [23]. Compared to these two studies, the present work includes a larger and more diverse population consisting of over 1000 patients—a far greater sample size with very broad racial diversity that points to the increased generalizability of our findings. In addition, our analysis was able to provide the vessel-related metrics by anatomic location (1 mm patch, 6 mm patch, and inner ring), a strategy that may have enabled the detection of more subtle differences in macular circulation.

One interesting consideration regarding FAZ-related and vessel-related metrics is their dependence on age and sex. Previous studies with normal subjects have found that VD-related measurements decrease with increasing age, and that FAZ area is larger in females than in males, which are consistent with the findings of this study [10,25,26]. Regarding sex, we found no statistical difference in mean VD between males and females. However, we detected decreased PD and VD in the 1 mm patch in females compared to males.

It is still unclear why some people do not develop DR despite a long duration of diabetes. Several studies have proposed possible mechanisms; the Joslin 50-Year Medalist Study challenged the notion that duration of diabetes is a strong predictor for retinopathy and indicated that a substantial proportion of diabetics are protected from advanced diabetic vascular complications [15]. Cabrera et al. hypothesized that these protected individuals are “extreme phenotype” cases of no DR, and that “protective factors”, perhaps genetic or environmental, could contribute to the slowing or prevention of retinopathy [27]. For instance, recent genome-wide association studies (GWAS) have identified genetic variants in angiogenesis and inflammatory pathways that may contribute to DR protection [28]. Furthermore, literature in other fields has similarly suggested that a subgroup of DM patients appear to be protected from other diabetic complications such as nephropathy and cardiovascular disease despite longer duration of DM [15]. In our analyses, we show that the FAZ area is largest in the medium cohort and significantly smaller in the long cohort. Furthermore, while FAZ perimeter is greater in the medium cohort compared to the short cohort, the long cohort and short cohort possess the same mean FAZ perimeter lengths. While these hypotheses were not explicitly tested for, our analyses support the recent studies that suggest that patients who have been DR-free for extended periods of time since their initial DM diagnosis may be more protected from developing DR in general.

This study has several limitations. First, it is retrospective in design. In addition, there is selection bias with this subject cohort because patients with a longer history of DM are more likely to develop DR, thus disqualifying them from the study. Furthermore, estimating diabetic duration based on patient report could have led to underestimating its length. Moreover, this study did not include normal subjects as a comparator group. The role of age as a potential confounder for FAZ area differences between the medium and long cohorts must be acknowledged, in light of prior work demonstrating age-related losses in perifoveal capillaries accounting for increased FAZ area in the elderly [29], but we believe that this is unlikely to account entirely for the FAZ differences between the medium and long cohorts since the long cohort group’s mean age was only 61.7 years. Finally, while we investigated the SCP slab, which was provided automatically by the OCTA software, we did not investigate the DCP and choriocapillaris (CC) slabs, which require manual acquisition. However, Durbin et al. suggest that the SCP was more practically effective than DCP as a diagnostic tool [23]. Additional studies to explore the association between OCTA parameters in the DCP and CC and duration of diabetes in patients with no DR are warranted.

## 5. Conclusions

Multiple OCTA parameters related to superficial perfusion and vascular density are negatively associated with DM disease duration in patients without clinically apparent DR. OCTA may be a useful tool for detecting deleterious preclinical microvascular changes in this patient population.

## Figures and Tables

**Table 1:** Baseline characteristics for patients in the short cohort (DM duration of 0–5 years), medium cohort (DM duration of 6–10 years), and long cohort (DM duration of more than 10 years).

		DM Duration		
	Short Cohort: 0–5 Years (*n* = 571)	Medium Cohort: 6–10 Years (*n* = 306)	Long Cohort: Over 10 Years (*n* = 241)	*p* Values
Age, year				*p* < 0.001 (SC vs. MC and SC vs. LC)
Mean (SD) [range]	54.6 (11.7) [15–84]	59.3 (10.2) [31–84]	61.7 (10.3) [22–85]	*p* = 0.012 (MC vs. LC)
Gender, No./total No. (%)				*p* = 0.072 (SC vs. MC)
Female	279/571 (48.9)	169/306 (55.2)	138/241 (57.3)	*p* = 0.029 (SC vs. LC)
Male	292/571 (51.1)	137/306 (44.8)	103/241 (42.7)	*p* = 0.634 (MC vs. LC)
Body mass index (BMI)				
Mean (SD)	31.32 (7.27)	30.37 (6.61)	30.62 (7.56)	*p* = 0.067
HbA1c, %				*p* = 0.012 (SC vs. MC)
Mean (SD) [range]	7.64 (2.05) [4.1–20.4]	7.76 (1.74) [4.4–15.1]	7.97 (2.27) [5.4–27.1]	*p* = 0.001 (SC vs. LC)
				*p* = 0.387 (MC vs. LC)
Hypertension				*p* < 0.001 (SC vs. MC and SC vs. LC)
No./total No. (%)	303/571 (53.1)	209/306 (68.3)	177/241 (73.4)	*p* = 0.190 (MC vs. LC)
Hyperlipidemia				*p* = 0.002 (SC vs. MC)
No./total No. (%)	299/571 (52.4)	194/306 (63.4)	156/241 (64.7)	*p* = 0.001 (SC vs. LC)
				*p* = 0.747 (MC vs. LC)
Diabetes treatment, No./total No. (%)				
Diet control	117/571 (20.5)	26/306 (8.5)	15/241 (6.2)	*p* < 0.001 (SC vs. MC and SC vs. LC)
Oral medicine	385/571 (67.4)	215/306 (70.3)	159/241 (66.0)	*p* = 0.158 (MC vs. LC)
Insulin use	69/571 (12.1)	65/306 (21.2)	67/241 (27.8)	

SC: short cohort; MC: medium cohort; LC: long cohort.

**Table 2:** Descriptive Statistics for Density Metrics and Foveal Avascular Zone (FAZ) Area in the Superficial Capillary Plexus (SCP) for the Three Diabetes Duration Groups.

	Perfusion Density			Vessel Density, mm^−1^			FAZ Area, mm^2^	FAZ Perimeter, mm	FAZ Circularity Index
Statistic	6 mm Patch	1 mm Patch	Inner Ring	6 mm Patch	1 mm Patch	Inner Ring			
Short cohort (SC):									
Mean (SD)	0.360 (0.040)	0.168 (0.054)	0.381 (0.036)	19.3 (1.9)	9.4 (3.0)	20.5 (2.0)	0.287 (0.112)	2.35 (0.51)	0.643 (0.110)
Medium cohort (MC):									
Mean (SD)	0.357 (0.038)	0.157 (0.053)	0.377 (0.034)	19.0 (1.9)	8.7 (2.9)	20.3 (2.0)	0.300 (0.118)	2.43 (0.55)	0.622 (0.112)
Long cohort (LC):									
Mean (SD)	0.348 (0.041)	0.160 (0.060)	0.369 (0.037)	18.6 (2.0)	8.9 (3.3)	19.7 (2.2)	0.272 (0.119)	2.35 (0.58)	0.609 (0.114)
*p* value	*p* = 0.119 (SC vs. MC)	*p* = 0.003 (SC vs. MC)	*p* = 0.172 (SC vs. MC)	*p* = 0.028 (SC vs. MC)	*p* = 0.001 (SC vs. MC)	*p* = 0.127 (SC vs. MC)	*p* = 0.105 (SC vs. MC)	*p* = 0.053	*p* = 0.002 (SC vs. MC)
	*p* = 0.027 (MC vs. LC)	*p* = 0.441 (MC vs. LC)	*p* = 0.008 (MC vs. LC)	*p* = 0.011 (MC vs. LC)	*p* = 0.458 (MC vs. LC)	*p* = 0.003 (MC vs. LC)	*p* = 0.004 (MC vs. LC)	(*)	*p* = 0.268 (MC vs. LC)
	*p* < 0.001 (SC vs. LC)	*p* = 0.062 (SC vs. LC)	*p* < 0.001 (SC vs. LC)	*p* < 0.001 (SC vs. LC)	*p* = 0.021 (SC vs. LC)	*p* < 0.001 (SC vs. LC)	*p* = 0.087 (SC vs. LC)	(*)	*p* < 0.001 (SC vs. LC)

Obvious outliers greater than 3 standard deviations from the mean were excluded. * The Kruskal–Wallis H test was used. In SPSS, if the H test is not statistically significant, the comparison between groups is no longer performed, resulting in one *p* value for FAZ perimeter.

**Table 3:** Standardized Coefficients for Association of Demographic and Clinical Variables with OCTA Density and Foveal Avascular Zone (FAZ) Parameters.

	Perfusion Density			Vessel Density, mm^−1^			FAZ Area, mm^2^	FAZ Perimeter, mm	FAZ Circularity Index
Variable	6 mm Patch	1 mm Patch	Inner Ring	6 mm Patch	1 mm Patch	Inner Ring			
Duration of diabetes									
0–5 years	ref	ref	ref	ref	ref	ref	ref	ref	ref
6–10 years	−0.025	−0.068	−0.014	−0.006	−0.077	0.000	0.061	0.070	−0.049
*p* value	0.447	0.038	0.673	0.860	0.018	0.989	0.069	0.037	0.145
10+ years	−0.065	−0.007	−0.071	−0.059	−0.014	−0.060	−0.045	−0.021	−0.061
*p* value	0.061	0.839	0.036	0.075	0.672	0.075	0.193	0.550	0.076
Age	−0.212	−0.153	−0.261	−0.349	−0.164	−0.336	−0.091	−0.006	−0.201
*p* value	<0.001	<0.001	<0.001	<0.001	<0.001	<0.001	0.008	0.866	<0.001
Gender	0.012	0.200	0.007	0.034	0.213	0.006	−0.209	−0.185	−0.024
*p* value	0.681	<0.001	0.819	0.235	<0.001	0.843	<0.001	<0.001	0.419
BMI	0.005	0.085	0.000	0.056	0.111	0.051	−0.057	−0.092	0.075
*p* value	0.865	0.005	0.995	0.057	<0.001	0.087	0.065	0.004	0.016
HbA1c	−0.014	−0.029	−0.030	−0.037	−0.029	−0.027	−0.002	0.003	−0.013
*p* value	0.668	0.356	0.339	0.229	0.349	0.375	0.957	0.915	0.685
Hypertension	−0.082	−0.035	−0.062	−0.004	−0.021	0.020	−0.007	0.021	−0.012
*p* value	0.010	0.264	0.050	0.897	0.501	0.511	0.832	0.526	0.705
Hyperlipidemia	0.059	0.021	0.050	0.014	0.005	0.005	0.021	0.012	0.012
*p* value	0.051	0.491	0.096	0.638	0.855	0.875	0.495	0.687	0.692
Treatment of diabetes									
Oral medicine	0.020	0.002	0.016	−0.004	−0.002	−0.006	−0.021	−0.007	−0.002
*p* value	0.632	0.952	0.703	0.915	0.954	0.874	0.619	0.870	0.971
Insulin use	−0.004	−0.004	−0.001	−0.030	−0.010	−0.025	−0.016	0.006	−0.023
*p* value	0.937	0.931	0.979	0.482	0.824	0.552	0.727	0.891	0.602

## Data Availability

The data presented in this study are available on request from the corresponding author. The data are not publicly available due to institutional regulations.
